# Incorporation of bioactive glass-ceramic into coconut oil for remineralization of incipient carious lesions

**DOI:** 10.1590/0103-6440202305636

**Published:** 2023-12-22

**Authors:** Jessica Dantas Abreu, Stéphanie de Oliveira Silva, Ayodele Alves Amorim, Eduardo José Soares, Rocio Geng-Vivanco, Carolina Noronha Ferraz de Arruda, Fernanda de Carvalho Panzeri Pires-de-Souza

**Affiliations:** 1 Department of Pediatric Dentistry, Ribeirão Preto School of Dentistry, University of São Paulo, 14040-904 Ribeirão Preto, Brazil.; 2 Department of Dental Materials and Prosthodontics, Ribeirão Preto School of Dentistry, University of São Paulo, 14040-904 Ribeirão Preto, Brazil.; 3 Department of Prosthodontics, School of Dentistry, Rio de Janeiro State University(UERJ), 20551-030, Rio de Janeiro, Brazil

**Keywords:** Dental caries, Coconut oil, Biosilicate, Glass-ceramic, Tooth remineralization

## Abstract

This study evaluated the efficacy of incorporating different concentrations of bioactive glass-ceramic (Biosilicate) into coconut oil on the remineralizing potential and surface roughness of white spot lesions. Fragments (6 x 6 x 2mm) of bovine teeth were sectioned and initial microhardness (KHN) and surface roughness (Ra) readings were obtained. The samples were submitted to cariogenic challenge to form white spot lesions and were separated into six groups (n=13): 1) Artificial Saliva (AS); 2) Coconut Oil (CO); 3) CO+2% Biosilicate (CO+2%Bio); 4) CO+5% Biosilicate (CO+5%Bio); 5) 2% Biosilicate Suspension (2% Bio) and 6) 5% Biosilicate Suspension (5% Bio). The treatments for 1 cycle/day were: immersion into the treatments for 5 minutes, rinsing in distilled water, and storage in artificial saliva at 37ºC. After 14 days, KHN and Ra readings were taken. The surface roughness alteration ((Ra) was analyzed (Kruskal-Wallis, Dunn’s post-test, p<0.05). CO+2%Bio had higher (p = 0.0013) (Ra followed by CO+5%Bio (p = 0.0244) than AS. The relative KHN and remineralization potential were analyzed (ANOVA, Tukey, p<0.05), and 5% Bio treatment presented a higher relative microhardness than all other groups (p>0.05). The remineralizing potential of all the treatments was similar (p > .05). When Biosilicate was added, the pH of the suspensions increased and the alkaline pH remained during the analysis. Biosilicate suspension is more efficient than the incorporation of particles into coconut oil at white spot lesion treatment. In addition to the benefits that coconut oil and Biosilicate present separately, their association can enhance the remineralizing potential of Biosilicate.

## Resumo

Este estudo avaliou a eficácia da incorporação de diferentes concentrações de vitrocerâmica bioativa (biosilicato) ao óleo de coco no potencial remineralizante e na rugosidade superficial de lesões de manchas brancas. Fragmentos (6 x 6 x 2mm) de dentes bovinos foram seccionados e as leituras iniciais de microdureza (KHN) e rugosidade superficial (Ra) foram obtidas. As amostras foram submetidas ao desafio cariogênico para formação de lesões de manchas brancas e foram separadas em seis grupos (n=13): 1) Saliva Artificial (AS); 2) Óleo de Coco (CO); 3) CO+2% Biosilicato (CO+2%Bio); 4) CO+5% Biosilicato (CO+5%Bio); 5) Suspensão de Biosilicato 2% (2% Bio) e 6) Suspensão de Biosilicato 5% (5% Bio). Os tratamentos de 1 ciclo/dia foram: imersão nos tratamentos por 5 minutos, enxágue em água destilada e armazenamento em saliva artificial a 37ºC. Após 14 dias, foram feitas as leituras de KHN e Ra. A alteração da rugosidade superficial ((Ra) foi analisada (Kruskal-Wallis, pós-teste de Dunn, p<0,05). CO+2%Bio apresentou maior (p = 0,0013) (Ra seguido de CO+5%Bio (p = 0,0244) do que AS. O KHN relativo e o potencial de remineralização foram analisados (ANOVA, Tukey, p<0,05), e o tratamento 5% Bio apresentou uma microdureza relativa maior do que todos os outros grupos (p>0,05). A suspensão de biosilicato é mais eficiente que a incorporação de partículas ao óleo de coco no tratamento de lesões de mancha branca. Além dos benefícios que o óleo de coco e o Biosilicato apresentam separadamente, sua associação pode amplificar o potencial remineralizante do Biosilicato.

## Introduction

Dental caries is a multifactorial disease with slow progression, which involves a dysbiosis of the microbial community due to the increase of acidogenic bacteria, a consequence of different factors such as excessive and frequent intake of sugars in the diet, salivary flow and composition, oral hygiene, fluoride exposure, in addition to behavioral and socioeconomic factors ^1^. White spot lesions can be considered the first sign of dental caries and exhibit increased porosity and could present a rough surface ^2^ ^,^ ^3^, however, at this stage the caries disease can be reversed ^4^. Therefore, well-established hygiene and prevention strategies are necessary to reverse this situation and achieve oral health.

The removal of biofilm through oral hygiene is the most efficient method to prevent caries disease. Fluoride-based products, such as toothpaste, are the most commonly used for this purpose ^5^. These products form F^-^ reservoirs on the enamel surface in the form of calcium fluoride crystals (CaF_2_) or fluorapatite ^6^, slowing down the demineralization and promoting remineralization of incipient lesions ^7^ ^,^ ^8^. However, nowadays, alternative protocols regarding natural products are in focus due to their simple use, as elaborate processes are not required to formulate or obtain the agent, minimal side effects, and low cost.

Biosilicate, a bioactive glass-ceramic characterized by fully crystallized particles, has been developed to merge the mechanical bioactivity of glass with the strength of glass ceramics ^9^. It boasts antimicrobial properties and exhibits potential for remineralizing dental structures, along with demonstrated effectiveness in bone regeneration ^10^. Biosilicate is known to interact with bodily fluids, forming a layer of hydroxycarbonate apatite (HCA). Additionally, studies have underscored its capability to promote the remineralization of erosive and carious lesions ^11^ and its role in remineralizing and sealing dentinal tubules, which contributes to the reduction of dentin hypersensitivity ^12^. The presence of fluids, as found in the oral cavity, further enhances Biosilicate's capacity to elevate local pH, thus fostering oral health maintenance ^13^.

Given Biosilicate's strong reactivity in the presence of fluids and its favorable impact on oral pH regulation ^9^, coconut oil has emerged as a promising option for preserving these valuable attributes of Biosilicate. Beyond serving as a carrier for Biosilicate, coconut oil, derived from Cocos nucifera, possesses notable properties conducive to oral health preservation ^13^ ^,^ ^14^. Coconut oil is endowed with anti-inflammatory, antibacterial, and antiviral properties ^15^. Its composition comprises 92% saturated fatty acids, with lauric acid accounting for 49% ^16^. Lauric acid exerts its effects against both gram-positive and gram-negative bacteria, as well as lipid-coated viruses, via physicochemical mechanisms ^16^. Consequently, coconut oil offers a viable avenue for incorporating active constituents and subsequently releasing them into the oral cavity ^17^. It is worth noting, however, that fluoride cannot be dissolved into coconut oil ^18^.

This study evaluated the efficacy of the incorporation of different concentrations of Biosilicate into coconut oil regarding the surface roughness and remineralizing effect of induced enamel white spot lesions. The hypotheses were that the incorporation of Biosilicate into coconut oil would remineralize the white spot lesions and, consequently, prevent or reduce the change in surface roughness on dental enamel.

## Materials and methods

### Sample preparation

Seventy-eight bovine teeth fragments (6 x 6 x 2 mm) were obtained using a low-speed diamond disc under water cooling in a metallographic cutter (Isomet 1000, Isomet, Buehler, Lake Bluff, IL, EUA). The enamel surface was flattened under refrigeration, with SiC sandpaper in decreasing granulations of 600 and 1200-grit, to standardize the surface roughness (above 0.2 μm). The sample size (n = 13) was calculated based on a pilot study, comparing means of microhardness, and using www.openepi.com, with a 95 % confidence interval and power of 80 %.

### Evaluated properties

###  Surface roughness analysis 

Surface roughness readings were performed before (initial readings) and after (final readings) the treatments. At each time of analysis, three readings were performed on each sample using a rugosimeter (Surfcorder SE 1700, Kosakalab, Tokyo, Japan): in the middle of the sample, 1 mm to the left, and 1 mm to the right. The mean of these three readings was considered as the surface roughness value. The surface roughness alteration was calculated using the formula:

$$Ra\;=\;{Ra}_{f}\;-\;{Ra}_{i}$$

Where, Ra_i_ is the initial surface roughness value and Ra_f,_ is the final one.

###  Microhardness analysis 

In the Knoop microhardness analysis (Micro Hardness Tester HMV-2, Shimadzu, Tokyo, Japan), a statical vertical load of 25 g was applied for 5 seconds. Microhardness readings were performed before the cariogenic challenge (initial reading), after the cariogenic challenge, and after the treatments. Similar to the surface roughness readings, at each time of analysis, three measurements were done: in the middle of the sample, 1 mm to the right, and 1 mm to the left. The mean of the three readings was considered as the microhardness value.

To calculate the relative microhardness, the following formula was used:

$$KHNr=\;({KHN}_{f}\;-\;{KHN}_{i})/{KHN}_{i}$$

Where, KHN_i_ is the initial microhardness value and KHN_f,_ is the final one.

The remineralizing potential of each treatment was also calculated using the formula:

$$RP(\%)\;=\;({KHN}_{f}\;-{\;KHN}_{c})/({KHN}_{i}\;-\;{KHN}_{c})\;x\;100$$

Where KHN_f_ is the final reading; KHN_i,_ is the initial one, and KHN_c_ is the reading performed after the cariogenic challenge.

###  Cariogenic challenge 

All the fragments were submitted to cariogenic challenge to produce white spot lesions. The dentin surfaces were protected with acid-resistant nail varnish (Colorama, L’Oréal Brazil, Rio de Janeiro, RJ, Brazil) and fixed in the bottom of the vial used. The fragments were then covered with 1.5 mL of 6 % carboxymethylcellulose demineralizing gel at a pH of 4.6 and stored at 4 ºC for 12 h ^19^. Subsequently, 1.5 mL of 0,1 M lactic acid (pH = 4.6) adjusted with 10 M NaOH was poured over the specimens, which were incubated for 14 days at 37 ºC ^19^.

At the end of the cariogenic challenge, the specimens were rinsed with distilled water, dried with absorbent paper, and stored in Eppendorf tubes filled with artificial saliva at 37 ºC. The fragments were separated into six groups (n = 13) and placed in petri plates according to the treatments they were submitted.

### Protocols

The pH of the proposed protocols (Table 1) was measured using a digital pH meter (Kasvi model K39-2014B, Paraná, Brazil). For control groups (artificial saliva and coconut oil), only initial measurements were performed. For the other groups, the measures were done after 5, 15, and 25 minutes of addition of Biosilicate.

**Table 1:** Distribution of the experimental groups.

Group	Treatment
AS	Artificial saliva
CO	Coconut oil
CO+2% Bio	Coconut oil + 2% Biosilicate
CO+5% Bio	Coconut oil + 5% Biosilicate
2% Bio	2% Biosilicate suspension
5% Bio	5% Biosilicate suspension

Artificial saliva was prepared with 0.1665 g of calcium chloride, 0.133 g of monosodium phosphate, 11.184 g of potassium chloride, 0.02 g of sodium azide, and 2.4228 g of Tris buffer; diluted in 1 L of deionized water ^20^. This solution was stored in an appropriate bottle, protected from light. No preparation was required for the coconut oil (Copra, Copra Indústria Alimentícia, Alagoas, Brazil) applied alone.

Biosilicate was used in concentrations of 2% and 5% (wt%). To prepare the suspensions in distilled water, 30 mL of distilled water were mixed with 0.006 g and 0.015 g of Biosilicate particles, resulting in 2% and 5% suspensions, respectively, based on the calculated mass (Density of water is 1 g/mL).

For the suspensions in coconut oil, considering the high density (0.92 g/mL) of this vegetable oil and that Biosilicate needs water to react, 0.99 mL of Tween 20 (Sigma-Aldrich, Darmstadt, Germany) and 20 mL of artificial saliva were added to 30 mL of coconut oil. Then, 0.33 g and 0.78 g of Biosilicate particles were added to obtain 2% and 5% suspensions, respectively. In both suspensions (water and coconut oil-based), the Biosilicate particles were added immediately before application. All the solutions were prepared immediately before use.

Each solution/suspension (30mL) was dispensed into the Petri plates and the samples were immersed for 5 minutes under shaking (150 rpm). They were then rinsed with distilled water for 1 minute and stored in artificial saliva at 37 °C until the next day. This cycle was performed once a day for 14 days. For samples immersed in saliva, they remained in contact with the saliva throughout the 14 days.

### Statistical analysis

Initially, data were tested for normality using the Shapiro-Wilk test. The data distribution was considered non-normal for surface roughness, and normal regarding the relative microhardness and remineralizing potential. Thus, the surface roughness data were analyzed using Kruskal-Wallis and Dunn’s test; and the microhardness and remineralizing potential by one-way ANOVA and Tukey’s test, all with 95 % significance level.

## Results

### Surface roughness analysis

**Figure 1:**
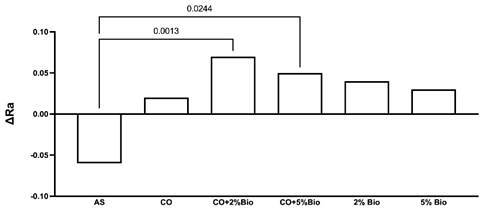
Comparison of surface roughness alteration among the groups.

A comparison of the surface roughness alteration mean values is presented in Figure 1. CO+2%Bio and CO+5%Bio presented higher (p < .05) surface roughness alteration than AS, with no difference (p > .05) between them. All the other groups presented no significant differences (p > .05).

### Microhardness analysis

**Figure 2:**
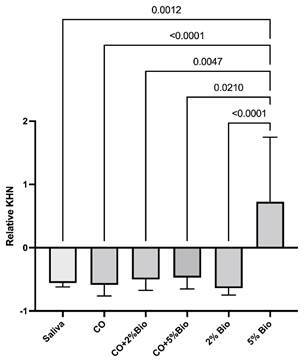
Comparison of the relative microhardness values among the groups.

A comparison of the relative microhardness mean values is shown in Figure 2. 5% Bio resulted in higher relative microhardness than all the other groups (p < .05), which presented no significant differences between them (p > .05).

A comparison of the remineralizing potential mean values can be seen in Table 2. The remineralizing potential of all the treatments was similar (p > .05).

**Table 2:** Mean comparison (standard deviation) for the remineralizing potential (%) of the treatments (one-way ANOVA, Tukey, p < 0.05).

Treatment	Remineralizing potential (%)
AS	24 (7)
CO	28 (18)
CO+2% Bio	38 (17)
CO+5% Bio	38 (21)
2% Bio	25 (9)
5% Bio	26 (12)

For all comparisons, p > .05

### Dynamic pH evaluation

**Figure 3:**
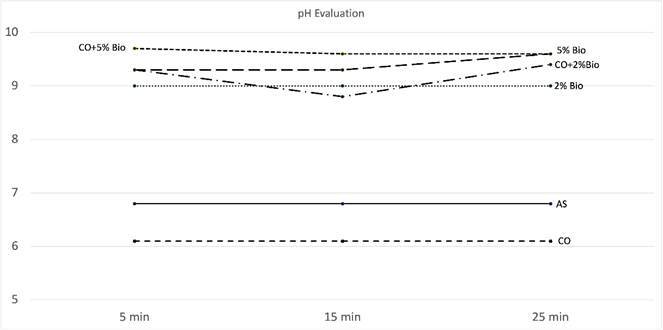
pH values over time for the proposed treatments.

pH values of the proposed treatments are described in Figure 3. The addition of Biosilicate particles immediately increased the pH of the suspensions and the alkaline pH remained during all the analysis.

## Discussion

This study aimed to evaluate the effect of different concentrations of Biosilicate (2 % and 5 %) incorporated into coconut oil on the remineralizing potential and surface roughness of enamel surfaces with white spot lesions. The hypothesis tested was that the incorporation of Biosilicate into coconut oil would remineralize the white spot lesions and preserve its surface roughness. Based on the results found, regarding microhardness, the hypothesis was rejected since the incorporation of Biosilicate into coconut oil resulted in a similar remineralizing potential to artificial saliva.

In the present study, artificial saliva showed similar relative microhardness values and remineralizing potential to the experimental treatments, except for 5% Bio which showed the highest relative microhardness (p < .05). Artificial saliva was considered as a control protocol in the present study as it is the most important biological factor for dental caries resistance ^21^. Its buffering capacity helps maintain a relatively neutral pH in the oral cavity that protects teeth and oral mucosa ^22^. In addition, salivary components contribute to the formation of the acquired pellicle covering the tooth surfaces ^23^, which acts as a natural barrier to prevent direct contact with acids and modulates the calcium, phosphate, and fluoride concentration on the surface ^24^.

Coconut oil is high in saturated fatty acids, mostly lauric acid ^25^. The lauric acid is a medium-chain fatty acid that in contact with enzymes is converted into monolaurin. Coconut oil has a high saponification value ^26^. The lauric acid reacts with sodium hydroxide in saliva to form sodium laurate which is responsible for its cleansing action and prevents plaque adhesion and accumulation ^25^. In the present study, the enamel demineralization was performed without microorganisms, so there was no plaque on the enamel surface. However, coconut oil could have had a cleaning ability, allowing direct contact of the enamel with the saliva ^27^.

Additionally, previous studies demonstrated that vegetable oils can modulate the composition and ultrastructure of the acquired pellicle making it even more resistant to acid challenges ^28^ ^,^ ^29^. Besides, coconut oil can remineralize the subsurface of the dental enamel ^30^ due to the high bioavailability of calcium in the coconut ^15^ ^,^ ^31^.

In this current research, we examined the potential of coconut oil, both alone and in combination with Biosilicate, in promoting remineralization. Our findings indicated that coconut oil exhibited remineralizing properties and yielded microhardness values similar to those observed with artificial saliva.

It is plausible that coconut oil operates in conjunction with saliva, facilitating a chemical reaction between the lauric acid present in coconut oil and the sodium hydroxide in saliva. This reaction likely results in the formation of sodium laureate, which is responsible for the cleansing effect on the tooth surface ^31^. This mechanism enhances the proximity of coconut oil to the tooth enamel, enabling interaction with the salivary components in the oral environment.

However, it is essential to note that the study did not permit sufficient time to fully explore the extent of coconut oil's contribution to the enamel remineralization process. Therefore, the results obtained in this investigation primarily underscore the role of saliva ^31^ and ^32^ in promoting remineralization. Further research is required to comprehensively elucidate the specific impact of coconut oil on this process.

Biosilicate has shown efficacy in reducing dentin hypersensitivity and controlling caries and erosive lesions ^11^ ^,^ ^31^ ^)^ due to its ability to remineralize the tooth structure and occlude the dentinal tubules ^12^ ^,^ ^32^ ^,^ ^33^. It forms HCA on hard tissues. Initially, in contact with fluids, the Biosilicate particles are dissolved, and alkaline ions are released producing a rapid increase in the local pH, as seen in Figure 3, where all the treatments with Biosilicate resulted in pH above 8.8. In an alkaline environment, silanols are formed to develop a silica gel layer on the tooth surface that stimulates the ionic exchange between the bioactive glass and the environment. Calcium and phosphate ions provided by the Biosilicate diffuse and form an amorphous calcium phosphate layer on the silica gel that is then crystallized into hydroxycarbonate apatite ^9^.

In previous studies ^11^ ^,^ ^12^ ^,^ ^31^ ^,^ ^32^ ^,^ ^33^ the concentration of Biosilicate tested was 10 % in suspension. To our knowledge, there is no previous evidence in the literature evaluating the effect of lower concentration of Biosilicate, as we tested in the present study. Considering our results, the concentration of Biosilicate is relevant for the remineralization of white spot lesions. 5% Bio presented higher relative microhardness than all the other groups (p < .05).

To saponify vegetable oils, an alkaline solution is required ^34^ ^,^ ^35^. The addition of Biosilicate could have boosted the saponification of the coconut oil resulting in higher cleaning efficiency. However, in the present study, the incorporation of Biosilicate into coconut oil did not increase the microhardness of the enamel with white spot lesions. Those groups presented similar remineralizing potential and relative microhardness than the other groups, except for 5% Bio (p < .05). Biosilicate needs time to interact with the dental substrates, and our results are justified by the delayed dissolution of Biosilicate particles in saliva due to the presence of coconut oil. The oil decreases the wettability of the saliva on the Biosilicate particles, and it would be necessary more time for the complete dissolution of this biomaterial. Conversely, 5 % Biosilicate suspension demonstrated the highest relative microhardness probably because Biosilicate particles were previously mixed with distilled water, facilitating their contact with the enamel surface. Regarding the surface roughness alteration, the hypothesis was also rejected, since 2 % and 5 % Biosilicate associated with coconut oil produced a rougher surface than the control group (artificial saliva).

Before the treatment, the samples were submitted to cariogenic challenge to produce white spot lesions. The demineralization process increases the porosity of the enamel surface, and consequently, the surface roughness is increased ^36^. As observed in the microhardness results, even after the treatment, full remineralization was not achieved, so probably the surface would still be porous. As mentioned before, the application time of the coconut oil was not sufficient for enamel remineralization, and it prevented the dissolution of the Biosilicate particles. Moreover, a higher concentration of Biosilicate particles would be required. Despite that, the surface roughness alteration values did not reach the critical limit to promote dental biofilm retention (≥ 0,2 µm)^37^.

This in vitro study presented an alternative preventive strategy for a common clinical situation in children: Sugar intake, mainly between meals, that causes constant low pH. Nearly 50 percent of beverages consumed by developed countries are sugar-sweetened beverages ^38^, and the consumption of these beverages will keep rising at about 2-3 % annually ^39^. Even though saliva can neutralize acids ^35^, in low pH environments below pH 5.5, the phosphate and calcium concentrations in saliva are reduced and its buffer capacity decreases ^2^ ^,^ ^3^ ^,^ ^40^. Thus, in such cases, a treatment capable of rapidly increasing the pH and presenting antimicrobial properties could be an excellent alternative. Besides, if the treatment presented remineralizing potential, it could decrease mineral loss, even without performing oral hygiene with fluoride-based toothpaste.

The results of the present study are promising; however, further studies are necessary to consolidate this evidence. An *in situ* study would be interesting to evaluate the treatments in a real clinical condition.

So, it was concluded that the Biosilicate is efficient for the treatment of white spot lesions. However, when incorporated into coconut oil it has less efficacy than when applied in a suspension. Besides, the concentration of Biosilicate particles affects its remineralizing capacity.
